# Effects of Single Nucleotide Polymorphism Ala270Ser (rs316019) on the Function and Regulation of hOCT2

**DOI:** 10.3390/biom9100578

**Published:** 2019-10-07

**Authors:** Dominik Frenzel, Christina Köppen, Oliver Bolle Bauer, Uwe Karst, Rita Schröter, Mladen V. Tzvetkov, Giuliano Ciarimboli

**Affiliations:** 1 Medizinische Klinik D, Experimentelle Nephrologie, Universitätsklinikum Münster, 48149 Münster, Germany; dominik.frenzel@web.de (D.F.); ritas@uni-muenster.de (R.S.); 2 Institut für Anorganische und Analytische Chemie, Westfälische Wilhelms-Universität, 48149 Münster, Germany; Christina.koeppen@basf.com (C.K.); Bolle.bauer@gmx.de (O.B.B.); uk@uni-muenster.de (U.K.); 3 Institut für Pharmakologie, Abteilung Klinische Pharmakologie, Universitätsmedizin Greifswald, 17487 Greifswald, Germany; mladen.tzvetkov@uni-greifswald.de

**Keywords:** organic cation transporter 2, single nucleotide polymorphism, cisplatin, nephrotoxicity

## Abstract

The human organic cation transporter 2 (hOCT2) is highly expressed in proximal tubules of the kidneys, where it plays an important role in the secretion of organic cations. Since many drugs are organic cations, hOCT2 has relevant pharmacological implications. The hOCT2 gene is polymorphic, and the nonsynonymous single nucleotide polymorphism (SNP) causing the substitution of alanine at position 270 of the protein sequence with serine (Ala270Ser) is present with high frequency in the human population. Therefore, Ala270Ser has potentially important pharmacologic consequences. Here, we analyzed the transport properties and rapid regulation of hOCT2 wildtype and hOCT2 Ala270Ser expressed in human embryonic kidney cells using real-time uptake measurements. Moreover, we compared the expression of hOCT2 in the plasma membrane determined by biotinylation experiments and the cellular transport and toxicity of cisplatin measured by inductively coupled plasma mass spectrometry and a viability test, respectively. The transport characteristics and regulation of the wildtype and mutated hOCT2 were very similar. Interestingly, a higher affinity of hOCT2 Ala270Ser for creatinine was observed. Compared with hOCT2 wildtype, the plasma membrane expression, cisplatin transport, and cisplatin-associated toxicity of hOCT2 Ala270Ser were significantly lower. In conclusion, these findings suggest that Ala270Ser has subtle but important effects on hOCT2 function, which are probably difficult to detect in studies with patients.

## 1. Introduction

The human organic cation transporter 2 (*SLC22A2*/hOCT2) is highly expressed on the basolateral membrane of renal proximal tubules [1]. Here, it mediates the uptake of organic cations into the cells, which is the first step in their renal secretion. Endogenous substances such as creatinine, dopamine, histamine, and serotonin, and exogenous substances such important drugs such as cimetidine and metformin belong to the class of organic cations [2]. Some inorganic compounds such as platinum derivatives [3,4,5,6,7,8] and Cd^2+^ [9] are also substrates of hOCT2; thus, hOCT2 is important for the renal secretion of its substrates and for inadvertent toxic effects of chemotherapeutic drugs such as cisplatin-associated nephrotoxicity. The activity of hOCT2 is subjected to rapid regulation by several signaling pathways [10], which are able to change its transport characteristics.

Moreover, it has been demonstrated that hOCT2 is polymorphic [11]. Because of its high allele frequency of 12.7% in many populations [11], the single nucleotide polymorphism (SNP) rs316019 is of high interest. This SNP involves a change of G to T at cDNA position 808 (G808T), which results in the substitution of alanine to serine in codon 270 of the protein sequence (Ala270Ser), being potentially able to change renal drug secretion [11].

However, already at the time of the SNP identification, it was evident that the transport properties of hOCT2 Ala270Ser differ very subtly from those of wildtype transporter (hOCT2 WT) [11], making it very difficult to predict the functional and pharmacological consequences of this mutation. Discordant effects of the polymorphism on metformin uptake in vitro and on metformin pharmacokinetics in humans were reported (for an overview see Reference [12]).

Indeed, focusing on cisplatin, there are also discordant reports on the role of rs316019 on cisplatin side effects. While some studies have reported a protective role of rs316019 against cisplatin-induced nephrotoxicity [13,14] and ototoxicity [15], other studies could not confirm these findings [16].

In this work, we analyzed the transport characteristics of hOCT2 WT and hOCT2 Ala270Ser expressed in an in vitro system, which guarantees the same expression levels of these proteins. Moreover, using this system, we investigated cisplatin cellular toxicity and uptake, and hOCT2 expression in the plasma membrane.

## 2. Materials and Methods

### 2.1. Cell Culture

Human embryonic kidney (HEK293) cells stably overexpressing hOCT2 WT or hOCT2 Ala270Ser and empty vector transfected cells were generated using T-Rex^TM^ HEK293 cells and the Flp-In^TM^ system (Invitrogen, Karlsruhe, Germany) as described previously [17,18]. Experimental details can be found in Reference [18]. Culture and function of these cells were approved by the state government Landesumweltamt Nordrhein-Westfalen, Essen, Germany (no. 521.-M-1.14/00).

Cells were grown in Dulbecco’s Modified Eagle’s Medium (DMEM) (Biochrom, Berlin, Germany) containing 10% fetal bovine serum (FBS, Biochrom), 1% penicillin/streptomycin (Biochrom), 10% L-glutamine (Biochrom), and hygromycin B (50 µL/ 50 mL; Roche, Mannheim, Germany) at 37 °C and 8% CO_2_ in 25-cm^2^ cell culture flasks (Greiner, Frickenhausen, Germany). For functional measurements, cells were transferred to 96-well plates (Greiner). Cells from passages 5–35 were used in the experiments.

Immunofluorescence was used to show hOCT2 expression in hOCT2 cells and controls (see Supplementary Figures S1 and S2).

### 2.2. Transport Measurements

Measurement of hOCT2 function in HEK293 cells was performed using microfluorimetry with the fluorescent organic cation 4-(4-(dimethylamino)styryl)-N-methylpyridinium (ASP^+^) as the substrate, as described previously in detail [19,20,21]. As shown in previous work, this method allows a real-time measurement of the transporter function [19]. The initial rate of ASP^+^ uptake was calculated, as it reflects the activity of the transporter [10,19,22]. 

Briefly, ASP^+^ cellular fluorescence was measured in a microtiterplate reader (Tecan infinite m200; Tecan, Crailsheim, Germany) at 37 °C using an excitation wavelength of 450 nm and an emission of 590 nm. Before fluorescence measurements, cells confluently grown in 96-well plates were washed in ringer-like solution (in mmol/L: 145 NaCl, 1.6 K_2_HPO_4_, 0.4 KH_2_PO_4_, 5 D-glucose, 1 MgCl_2_, 1.3 calcium gluconate, pH 7.4). Cell fluorescence was measured before and after ASP^+^ addition to the cells. The kinetic characteristics of hOCT WT and hOCT2 Ala270Ser were investigated performing saturation experiments with increasing ASP^+^ concentrations. Non-hOCT2-mediated ASP^+^ uptake was evaluated in the presence of a huge amount (1 mM) of tetrapentylammonium (TPA^+^) and subtracted from the total ASP^+^ uptake in order to evaluate the ASP^+^ uptake mediated specifically by hOCT2, as shown already in References [19,20]. Transport kinetics were described by maximum velocity of transport (V_max_) and Michaelis constant (K_m_) which defines the concentration where 50 % of V_max_ is reached. V_max_ and K_m_ of ASP^+^ uptake were defined according to the Michaelis-Menten kinetics model, where Y is the fluorescence increase in arbitrary units (a.u.)/µg protein s^2^ and X is the ASP^+^ concentration in µM:

Y = Vmax*X/(Km + X)(1)

The characterization of ASP^+^ transport was performed using at least two independent hOCT2 WT and hOCT2 Ala270Ser clones. Moreover, the cellular uptake of 1 µM ASP^+^ was measured in the presence or absence (control experiments) of potential endogenous (creatinine, histamine, and serotonin) and exogenous (cimetidine, cisplatin, imipramine, metformin, thiamine, and tryptophan) uptake competitors at increasing concentrations, in order to measure their apparent affinity (IC_50_) towards hOCT2. The IC_50_ values describe the inhibitory concentration needed to reduce the uptake to 50 % of control experiments and are calculated according to the following equation:

Y = Bottom + (Top-Bottom)/(1+10 ^X-logIC50^) (2)

This equation is easy to use when the data clearly define the bottom and the top plateaus of the curve. In our experiments, top is defined as binding in the absence of any competitor (100%). In the cases where a bottom plateau is not clearly reached, the software used (GraphPadPrism, San Diego, CA, USA) fits it based on the overall shape of the data. 

All solutions were applied at 37 °C and pH 7.4.

Moreover, since it is known that the activity of hOCT2 can be regulated by several kinases, the effect of the Ala270Ser substitution on regulation by calmodulin and p56*^lck^* tyrosine kinase of hOCT2 was investigated. The regulation by these two pathways is conserved along all the OCTs [10,20,23]. To study the rapid regulation of hOCT2, cells were incubated for 10 min at 37 °C with calmidazolium (5 µM), a potent specific inhibitor of calmodulin [24], with aminogenistein (10 µM), a specific inhibitor of p56*^lck^* tyrosine kinase [25], or with ringer-like solution as a control. Regulators were dissolved in ethanol (calmidazolium) or DMSO (aminogenistein) at a concentration that had no impact on ASP^+^ transport. After incubation, fluorescence measurements were carried out with 1 µM ASP^+^.

### 2.3. Biotinylation of Cell Surface Proteins

Biotinylation of cell surface proteins was used to isolate plasma membrane proteins from HEK293 cells grown to confluency. The Pierce Cell Surface Protein Isolation Kit (Thermo Scientific, Rockfort, IL, USA) was used according to the instructions of the manufacturer. Cells were washed two times with Phosphate buffered saline (PBS) at 4 °C and incubated for 30 min with biotin at 4 °C. A quenching solution was used to terminate the reaction, and cells were washed and lysed with lysis buffer. After addition of a protease inhibitor (Roche Applied Science, Mannheim, Germany), cells were centrifuged and then incubated for 60 minutes on a NeutrAvidin column (Thermo Scientific). The columns were washed, and cell surface proteins were collected using sodium dodecyl sulfate-polyacrylamide gel electrophoresis (SDS-PAGE) buffer containing dithiothreitol (DTT). Samples for the measurement of total proteins were separated before incubation on the NetrAvidin column and resuspended in SDS-PAGE buffer. The samples were transferred to an SDS-PAGE gel (Mini-PROTEAN TGX GEL, Bio-Rad, Munich, Germany) together with electrophoresis buffer. Electrophoresis was performed for one hour at 100–160 V. The gel was then blotted for 1 hour at 100 V on a polyvinylidene difluoride (PVDF) membrane (Roche). The PVDF membrane was incubated for 5 min in 3% gelatin to block unspecific binding and incubated overnight with mouse anti hOCT2 antibody (kindly provided by Prof. Koepsell [10]) at a 1:250 dilution. The PDVF membrane was incubated for 45 min with horseradish peroxidase (HRP) coupled with goat-anti-mouse antibody (Dako, Hamburg, Germany) at a 1:5000 dilution and washed again. Immunoreactive bands were detected by enhanced chemiluminescence. 

### 2.4. Cytoviability Assay

The cytotoxicity of cisplatin was evaluated using a modified 3-(4,5-dimethylthiazol-2-yl)-2,5-diphenyltetrazoliumbromid (MTT) assay [26]. The HEK293 cells grown for 24 hours in 96-well plates were incubated for 10 min at 37 °C with different concentrations of cisplatin (Calbiochem, Merck Chemicals GmbH, Darmstadt, Germany) dissolved in a ringer-like solution. The cisplatin solution was then removed, and the cells were grown in fresh medium for 48 hours. Afterwards, the cells were incubated with 10 µL MTT (Sigma, Steinheim, Germany) solution containing 5 mg/mL of the dye for three hours at 37 °C. Then, MTT was removed, and the cells were lysed with a solution containing 10% (*w*/*v*) sodium dodecyl sulfate and 40% (*v*/*v*) dimethylformamide. After 90 min, absorption was measured at 570 nm in a multiplate reader (Tecan infinite m200, Tecan, Tecan Group Ltd., Crailsheim, Germany).

### 2.5. Inductively Coupled Plasma Mass Spectrometry

Intracellular cisplatin concentrations were determined using inductively coupled plasma mass spectrometry (ICP-MS), which provides information on the elemental concentration in samples [27,28], as described before [29,30]. To do this, cells were grown for five days on 6-well plates and incubated at 37 °C for 2 min with 2 mL of 100 µM cisplatin freshly prepared in a ringer-like solution. After incubation, cells were rapidly washed with ice-cold PBS and then hypotonically lysed using distilled water and sonication for 15 min. For the ICP-MS analysis, 100 µL of cell lysates together with 100 µL of rhodium standard (Rh 1000 µg/mL, Scpscience, Marktoberdorf, Germany) were mixed with 9.8 mL 2% HNO_3_. Standard platinum for ICP (Merck) was diluted in 2% HNO_3_, and linear regression was performed (r² = 0.998 and 0.999). Samples were analyzed using ICP-MS iCap QC, autosampler AccelaAS (Thermo Fisher Scientific, Bremen, Germany) and Qtegra ISDS software (Thermo Fisher Scientific). The protein content of lysed cells was determined by absorption measurements using PicoDrop Pico 1000 (PicoDrop, Saffron Walden, UK).

### 2.6. Statistics

Statistical analyses were carried out using GraphPad Prism version 5.03 (GraphPad Software, Inc., La Jolla, CA, USA). Kinetic parameters of ASP^+^ uptake were compared using the extra sum-of-squares F test. Regulation of transporter function was analyzed using analysis of variance (ANOVA) with Tukey post-hoc test. Other assays were analyzed by unpaired t-tests. Statistical significance level was set at *p* < 0.05. 

## 3. Results

### 3.1. Fluorescence Measurements

Fluorescence measurements of hOCT2-mediated ASP^+^ uptake were performed using HEK293 cells stably expressing WT hOCT2 (hOCT2 WT) or hOCT2 carrying the serine variant of the Ala270Ser polymorphism (hOCT2 Ala270Ser). The transfection system used allows a targeted integration of the expression plasmid in the same locus of the genome, ensuring homogeneous levels of transfected protein expression. 

**Figure 1:**
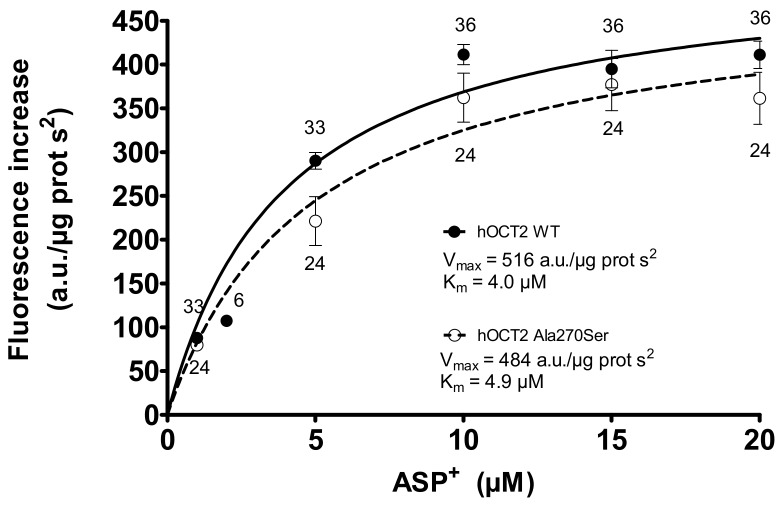
Specific ASP^+^ uptake mediated by the wildtype form of hOCT2 (hOCT2 WT, closed circles) and by hOCT2 Ala270Ser (open circles) in stably transfected HEK-cells as calculated by adding rising ASP^+^ concentrations to the cells and detecting the cellular fluorescence intensity. The specific uptake was calculated by subtracting the total uptake from the unspecific uptake determined in the presence of 1 mM TPA^+^. The number near the experimental points indicates the replicates measured in at least three independent experiments. Since both curves reached a plateau, it was possible to calculate K_m_ (4.0 ± 1.0 and 4.9 ± 1.6 µM for hOCT2 WT and hOCT2 Ala270Ser, respectively) and V_max_ (516 ± 23 and 484 ± 50 arbitrary units/µg protein s^2^ for hOCT2 WT and hOCT2 Ala270Ser, respectively) of ASP^+^ uptake. These values resulted not to be significantly different when compared using the extra sum-of-squares F test (GraphPad Prism).

Saturation experiments using these cells showed that the kinetic characteristics (K_m_ and V_max_) of ASP^+^ transport by hOCT2 WT and hOCT2 Ala270Ser are very similar (Figure 1).

**Figure 2:**
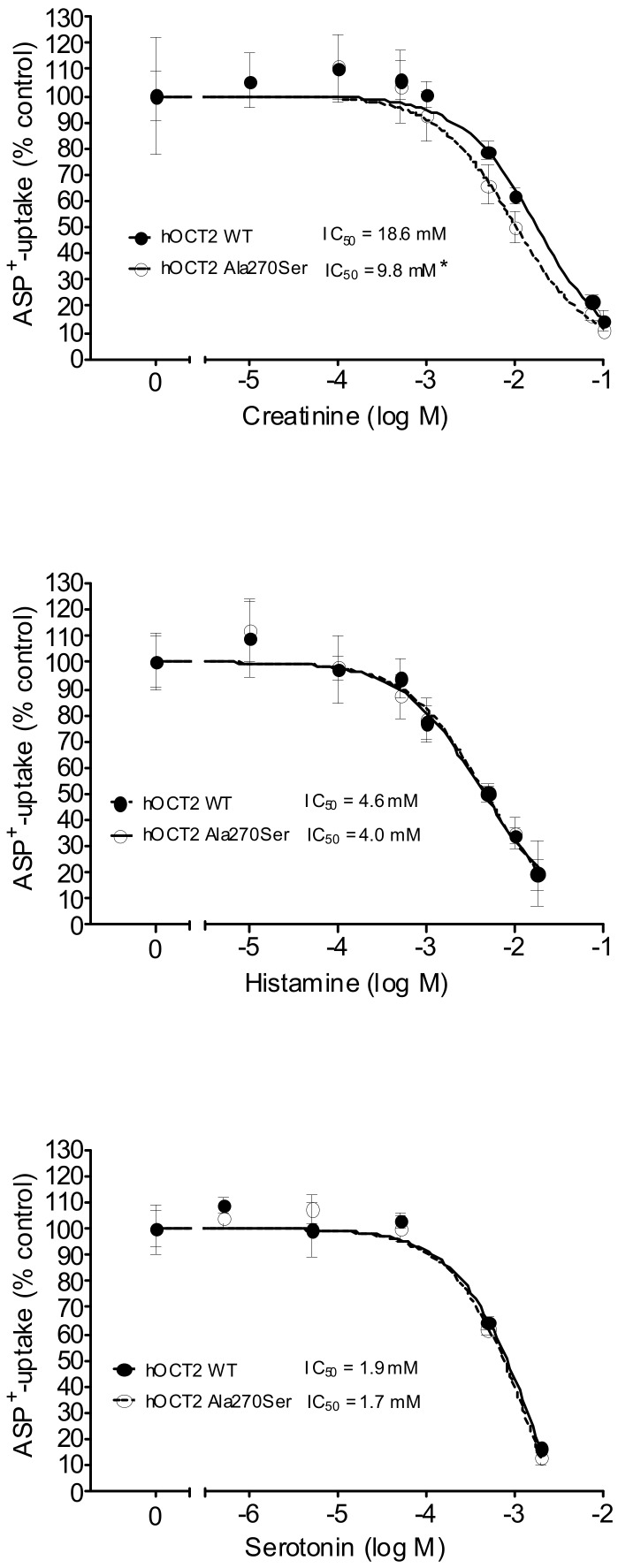
This figure shows the inhibition of ASP^+^ uptake (mean ± standard error of the mean (SEM)) by creatinine (upper panel, 6–72 replicates for each concentration), histamine (middle panel, 12–36), and serotonin (lower panel, 6–18) in hOCT2 WT (●, continued line) and hOCT2 Ala270Ser (○, dotted line) cells, measured in at least 3 independent experiments. For every substance, a concentration-dependent inhibition of ASP^+^ uptake was observed. The calculated IC_50_ values are also reported in the figures. The IC_50_ of creatinine was significantly different between hOCT2 WT and hOCT2 Ala270Ser (*) when compared using the extra sum-of-squares F test (GraphPad Prism), with the mutated transporter having a higher affinity for the substance.

The interaction of hOCT2 WT and hOCT2 Ala270Ser with several potential substrates of endogenous and exogenous origin was compared by competition experiments with ASP^+^ uptake. The endogenous substances creatinine, histamine, and serotonin displayed a low-affinity interaction with both transporters, as shown by the IC_50_ in the mM range (Figure 2). Only for creatinine, a small but significant difference between the IC_50_ measured in hOCT2 WT and hOCT2 Ala270Ser cells was detected, with the mutated form having a higher affinity for the substance.

**Figure 3:**
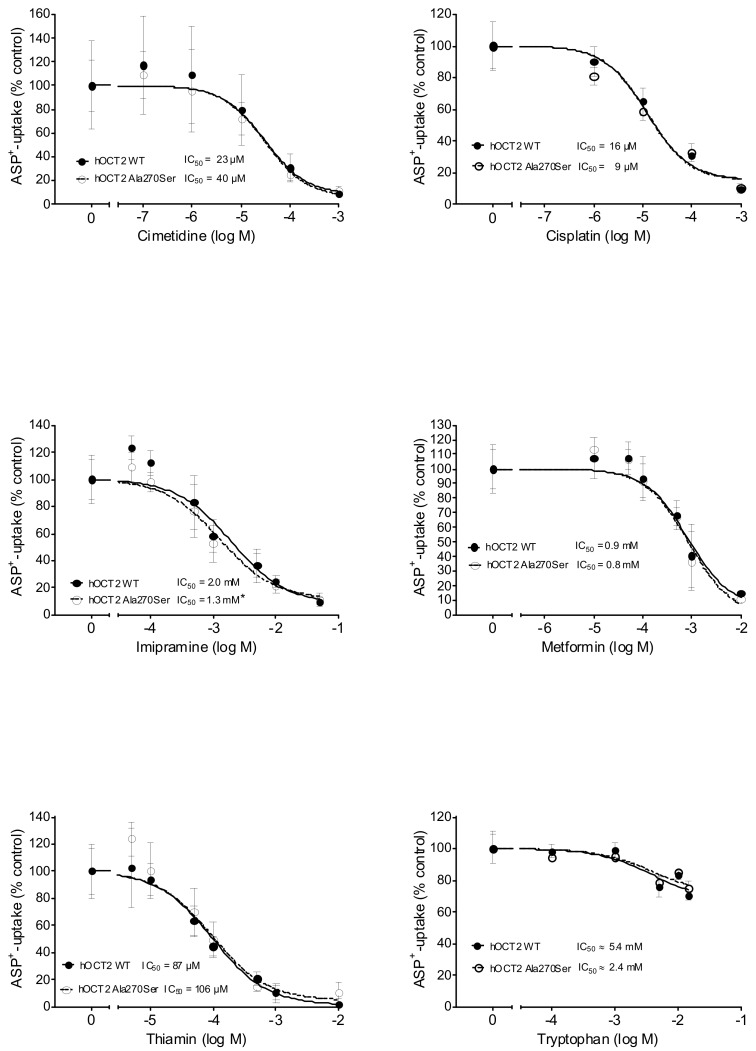
This figure shows the inhibition of ASP^+^ uptake (mean ± SEM) by cimetidine (upper left panel, 12–60 replicates for each concentration), cisplatin (upper right panel, 6–18 replicates), imipramine (middle left panel, 18–72 replicates), metformin (middle right panel, 18–54 replicates), thiamin (lower left panel, 12–54 replicates), and tryptophan (lower right panel, 6–12 replicates) in hOCT2 WT (●, continued line) and hOCT2 Ala270Ser (○, dotted line) cells, measured in at least 3 independent experiments. For every substance except tryptophan, a concentration-dependent inhibition of ASP^+^ uptake was observed. The calculated IC_50_ values are also reported in the figures. The IC_50_ of imipramine was significantly different between hOCT2 WT and hOCT2 Ala270Ser (*) when compared using the extra sum-of-squares F test (GraphPad Prism), with the mutated transporter showing a higher affinity for the substance.

The exogenous substances cimetidine, cisplatin, imipramine, metformin, and thiamin were able to inhibit the ASP^+^ uptake mediated by hOCT2 WT and hOCT2 Ala270Ser in a concentration-dependent manner (Figure 3), with the IC_50_ being in the micromolar range for cimetidine, cisplatin, and thiamine, and in the millimolar range for imipramine and metformin. For imipramine, a small but significant difference between the IC_50_ measured in hOCT2 WT and hOCT2 Ala270Ser cells was detected, with the mutated form having a higher affinity for the substance also in this case. Tryptophan was able to inhibit a small part of the ASP^+^ uptake only at very high concentrations. For this reason, the IC_50_ is an approximate value. Table 1 shows a summary of the IC_50_ values calculated for the different substances.

### 3.2. Regulation of ASP^+^ Uptake

**Figure 4:**
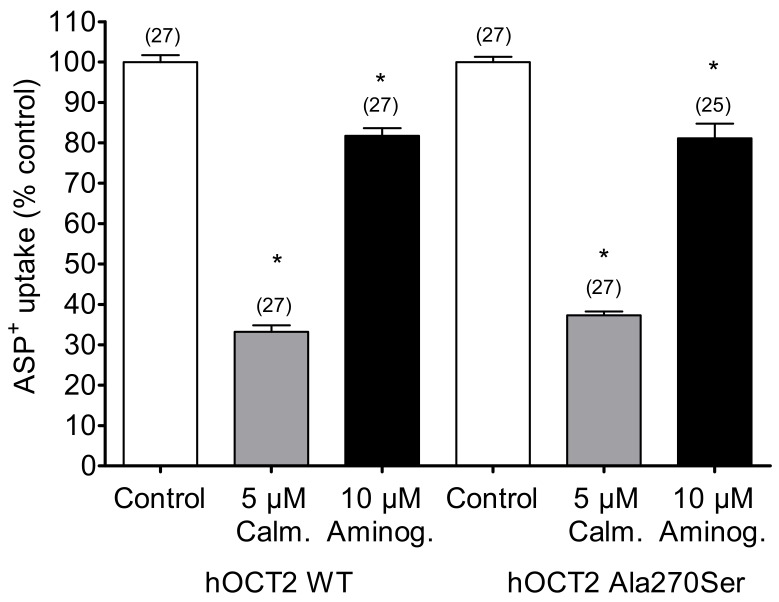
Regulation of hOCT2 WT- and hOCT2 Ala270Ser-mediated ASP^+^ uptake (mean ± SEM) under inhibition of calmodulin by 10 min incubation with five calmidazolium or under inhibition of p56*^lck^* tyrosine kinase by 10 min incubation with 10 µM aminogenistein. These maneuvers resulted in a strong significant inhibition (*, *p* < 0.05, ANOVA with Tukey post-hoc test) of transport in both cell lines, compared to that observed in control experiments. However, the degree of inhibition was not different in hOCT2 WT and hOCT2 Ala270Ser cells. The number of replicates measured in at least three independent experiments is shown on the top of the columns.

To examine whether the Ala270Ser SNP may alter the regulation of hOCT2, two common regulatory pathways (the calmodulin- and p56lck tyrosine kinase-associated regulation pathways) were analyzed in hOCT2 WT and hOCT2 Ala270Ser. The inhibition of the calcium calmodulin pathway by 5 µM calmidazolium reduced the transport of ASP+ similarly in both cell lines (Figure 4). Aminogenistein, an inhibitor of the p56lck tyrosine kinase pathway, had a similar inhibitory effect on both WT and Ala270Ser hOCT2 (Figure 4).

### 3.3. Cisplatin Toxicity in hOCT2 WT and hOCT2 Ala270Ser Cells

**Figure 5:**
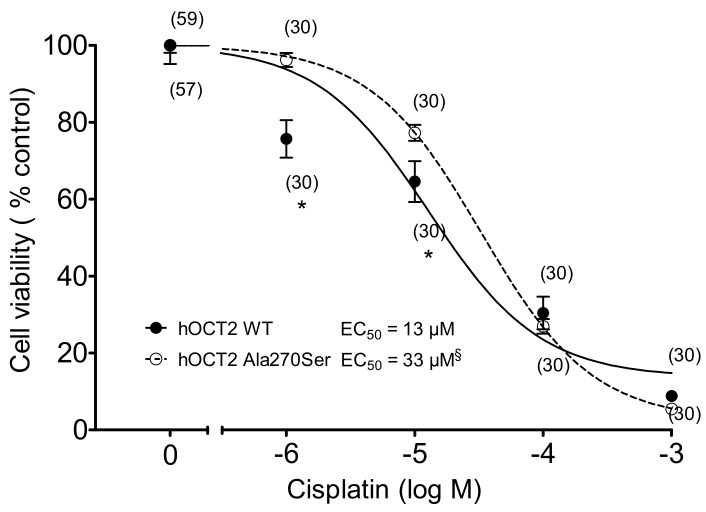
Cell toxicity induced by 10 min incubation with increasing cisplatin concentrations measured with an MTT assay (mean ± SEM) in hOCT2 WT (●, continued line) and hOCT2 Ala270Ser (○, dotted line) cells in at least three independent experiments. The number of replicates measured is shown close to the experimental points. A concentration-dependent cisplatin toxicity was observed. The calculated EC_50_ values are also reported in the figure. The EC_50_ of the cisplatin effect was significantly different between hOCT2 WT and hOCT2 Ala270Ser, when compared using the extra sum-of-squares F test (^§^, GraphPad Prism), being the mutated transporter more resistant against cisplatin toxicity. The difference was especially evident at low cisplatin concentrations (1 and 10 µM, * *p* < 0.05, unpaired t-test).

The sensitivity of hOCT2 WT and hOCT2 Ala270Ser cells to treatment with increasing concentrations of cisplatin was compared using a MTT assay. The concentrations of cisplatin, which gave 50% of the toxic effect (EC_50_), were 33 µM for hOCT2 Ala270Ser (logEC_50_ ± SEM = −4.48 ± 0.05, with 177 degrees of freedom, DF) versus 13 µM (logEC_50_ ± SEM = −4.89 ± 0.11, 175 DF) for hOCT2 WT (Figure 5). The difference was more evident at low cisplatin concentrations (1 and 10 µM). The difference resulted to be statistically significant (extra sum-of-squares F test (GraphPad Prism)). 

### 3.4. Cisplatin Cellular Accumulation Measured by Inductively Coupled Plasma MassSspectrometry

Since hOCT2 Ala270Ser cells are apparently more resistant against cisplatin toxicity, the intracellular concentrations of platinum were determined to test whether mutant and WT hOCT2 transport cisplatin differently into the cells. After incubation with 100 µM Cisplatin for 2 min, HEK293 cells were lysed, and intracellular platinum concentrations were measured using ICP-MS.

**Figure 6:**
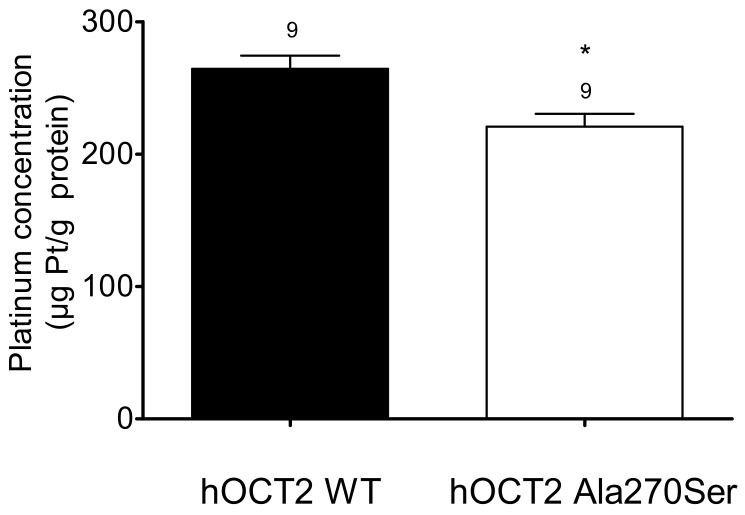
Cellular platinum concentrations (µg Pt/g protein, mean ± SEM) measured after 2 min incubation of hOCT2 WT (■) and hOCT2 Ala270Ser (□) cells with 100 µM cisplatin. The number of replicates measured in three independent experiments is shown above the column. hOCT2 WT cells accumulated significantly more platinum than hOCT2 Ala270Ser cells (*, *p* < 0.05, unpaired t-test).

There was a significantly higher concentration of platinum in hOCT2 WT cells than in hOCT2 Ala270Ser cells (264 ± 10 versus 221 ± 10 µg Pt/g protein, respectively) as shown in Figure 6. 

### 3.5. Expression of hOCT2 in the Plasma Membrane

**Figure 7:**
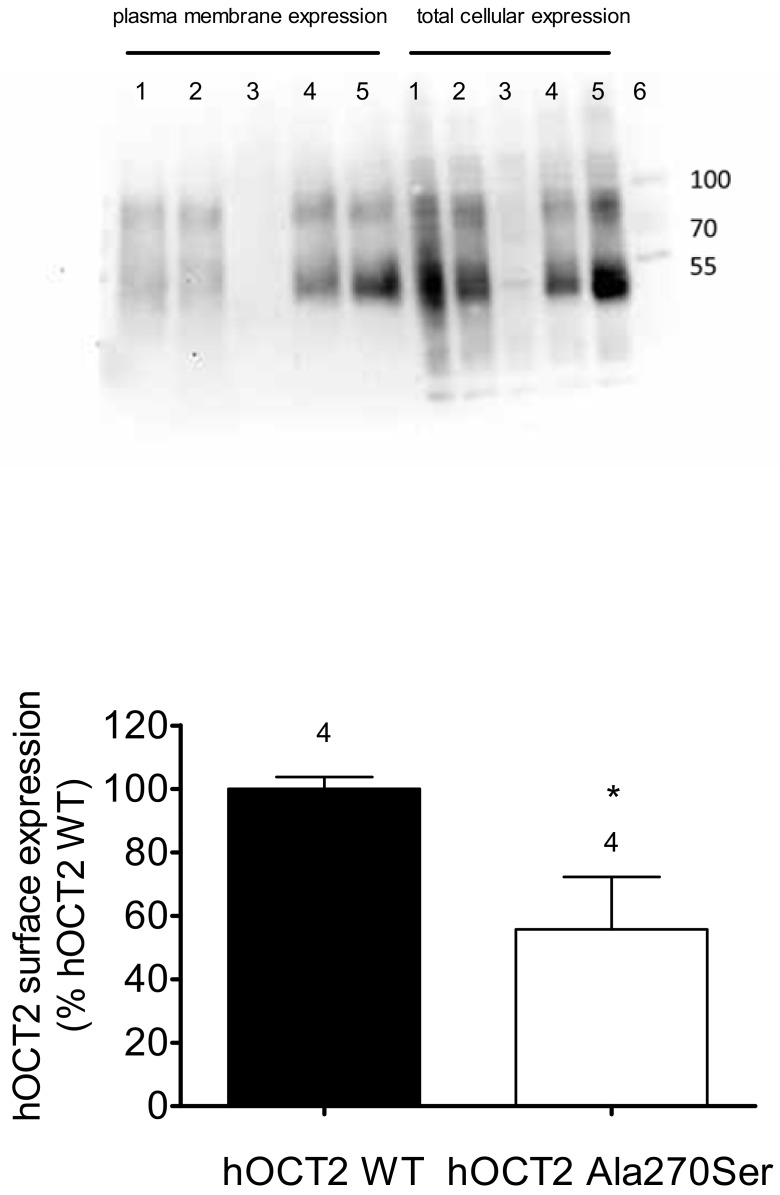
Investigation of hOCT2 expression in the plasma membrane by biotinylation experiments. The upper panel shows an example of Western blot analysis of hOCT2 expression in biotinylated fraction and total cell lysates. Lanes 1 and 2 contain samples from hOCT2 Ala270Ser cells, lanes 3 samples from cells transfected with empty vector, and lanes 4 and 5 samples from hOCT2 WT cells. Lane 6 contains molecular weight markers, whose molecular dimensions in kDa are indicated by the numbers on the right. The lower panel shows the quantification of four independent biotinylation experiments (as indicated by the numbers above the columns), where the ratio between band intensities in the biotinylated fractions and the total cellular lysates are represented as hOCT2 surface expression (% expression hOCT2 WT). hOCT2 WT cells expressed more hOCT2 in the plasma membrane than hOCT2 Ala270Ser cells (*, *p* < 0.05, unpaired t-test).

Since the hOCT2 Ala270Ser cells accumulate less platinum than the hOCT2 WT cells, but the apparent affinities of these cells for cisplatin are not different, the plasma membrane expression of hOCT2 in the two cell lines was evaluated by biotinylation experiments. As a measure of transporter expression in the plasma membrane, the ratio between total cellular expression and transporter expression in the biotinylated fraction was calculated. The upper panel of Figure 7 shows an example of Western blot analysis of biotinylated and total cellular fraction from hOCT2 Ala270Ser (lanes 1 and 2), hOCT2 WT (lanes 4 and 5), and empty vector transfected cells (lanes 3). In Western blot analysis, the hOCT2 signal consists in two different bands, corresponding to a glycosylated transporter form between 70 and 100 kiloDalton (kDa) and a non-glycosylated form at around 50 kDa, as already demonstrated [31]. 

As evident from the quantification in four independent surface biotinylation experiments, the expression of hOCT2 in the plasma membrane was significantly lower in hOCT2 Ala270Ser cells than in hOCT2 WT cells. No expression of hOCT2 was detected in cells stably transfected with the empty vector. 

This difference in membrane protein expression may potentially explain the reduced transport capacity of hOCT2 Ala270Ser compared with hOCT2 WT.

## 4. Discussion

Cisplatin is a widely used chemotherapeutic agent that has dramatically improved the outcome of testicular cancer [32,33]. One major side effect of cisplatin therapy is nephrotoxicity that is dose-limiting for patients [34]. Upon treatment, cisplatin accumulates in cells of the renal proximal tubules. Intracellular cisplatin targets the DNA and the cellular anti-oxidant defenses, decreasing the cell resistance against oxidative stress and disturbing DNA processing, thus causing cell apoptosis. This toxicity impairs the tubular functions and manifests as acute kidney injury (AKI), which can progress to chronic kidney disease. Up to around 20% of AKI-related deaths are associated with cisplatin-based chemotherapy [35,36]. Proximal tubule cells are able to accumulate cisplatin, as five-fold serum concentrations are found in these cells [37]. This fact suggests the involvement of a transporter, which is able to concentrate cisplatin in the cells. The uptake mediated by OCT2 is driven by the electrochemical potential of the substrate, and hOCT2 is described as a concentrative transporter [7]. It was shown that OCT2 is involved in cisplatin transport into proximal tubule cells [8,38] and critically mediates the development of cisplatin nephrotoxicity [3,4]. For these reasons, it can be assumed that hOCT2 is a transporter that is able to mediate the cisplatin accumulation in renal proximal tubules.

Even though multiple clinical studies suggest an ameliorative effect of rs316019 (SNP Ala270Ser in hOCT2) on cisplatin-induced nephrotoxicity [13,14,39], other studies did not find any differences between patients bearing rs316019 SNP or the wildtype transporter [16,40]. One study showed an even higher cisplatin nephrotoxicity for patients bearing the rs316019 SNP [41]. Moreover, no in vitro data on the effect of SNP rs316019 on cisplatin transport and toxicity have been published to date. As clinical data are inconsistent so far, the goal of this study was to assess the effects of the SNP rs316019 in hOCT2 on the interaction of cisplatin with hOCT2. Furthermore, the functional properties and regulation of hOCT2 WT and hOCT2 Ala270Ser were compared using HEK293 cells, where the hOCT2 WT or the hOCT2 Ala270Ser were expressed using a technique that results in the same level of transfected cDNA expression [17].

Using this expression system, ASP^+^ alone or together with different exogenous and endogenous substrates of hOCT2 were administered, in order to assess the kinetic characteristics (K_m_ and V_max_) of ASP^+^ uptake and the effects of these substrates on it. The K_m_ of hOCT2 WT and hOCT2 Ala270Ser for ASP^+^ were not different. Also, the V_max_ values were not statistically significant different. However, it has to be considered that a direct comparison of V_max_ values obtained using this fluorescence technique is difficult, since they are expressed in arbitrary units based on the emitted fluorescence. This fluorescence is dependent on the performance of the lamp, which is of course changing over time [10]. For most substrates (cimetidine, cisplatin, histamine, metformin, serotonin, thiamin, tryptophan) there was no difference between hOCT2 WT and hOCT2 Ala270Ser. For tryptophan, only high concentrations (>1 mM) were able to inhibit only a small part of the ASP^+^ uptake. Generally, the degree of inhibition was similar in hOCT2 WT and hOCT2 Ala270Ser cells, except for creatinine and imipramine. These two substances displayed a higher affinity for hOCT2 Ala270Ser (IC_50_: 9.8 mM and 1 mM for creatinine and imipramine, respectively) than hOCT2 WT (IC_50_: 18.6 mM and 2 mM for creatinine and imipramine, respectively). In other works, imipramine did not show a different interaction with hOCT2 WT and hOCT2 Ala270Ser [42]. Similarly, there are contradictory results in the literature concerning the power of interaction of different substrates with hOCT2 WT and hOCT2 Ala270Ser [11,42,43]. These discrepancies may be explained by different expression systems and transfection techniques used and also by the tracers used, because of the special structure of OCT binding pocket. This binding pocket is large and may contain more than one binding site for the substrates; moreover, the binding domains for different substrates may have a different degree of overlapping [44,45,46,47]. Nevertheless, the higher affinity of the endogenous substrate creatinine towards hOCT2 Ala270Ser than towards hOCT2 WT (IC_50_: 9.8 versus 18.6 mM, respectively) may be of clinical significance, since creatinine is often used to calculate renal function as estimated glomerular filtration rate (eGFR) [48]. A higher affinity towards hOCT2 could lead to a higher tubule secretion of creatinine, resulting in a delayed increase of serum creatinine levels as a sign of decreased kidney function. Consequently, renal injury would be detected at a later stage or would not be detected at all in patients expressing Ala270Ser genotype. In fact, results on the hOCT2 genotype effect on cisplatin treatment nephrotoxicity are discordant [13,16,49]. Therefore, in future studies, different markers of kidney function such as cystatin c should be used to monitor kidney function. Indeed, higher levels of cystatin c were measured in patients with wildtype hOCT2 in contrast to that observed in patients bearing Ala270Ser hOCT2 [14], showing a protection by hOCT2 Ala270Ser against cisplatin nephrotoxicity. 

Our in vitro studies with the MTT assay confirmed the protection conferred by the expression of hOCT2 Ala270Ser against cisplatin cellular toxicity compared to that observed in hOCT2 WT cells. It is well known that the activity of hOCT2 is regulated by several signaling pathways [10]. The polymorphic hOCT2 did not show any different regulation from the wildtype transporter, suggesting that this is not an explanation for different sensitivity against cisplatin. In ICP-MS studies a reduced cellular platinum concentration in hOCT2 Ala270Ser cells was revealed, suggesting that differences in toxicity were mediated by altered cisplatin uptake. The reason for this may be the lower plasma membrane expression of hOCT2 Ala270Ser compared with hOCT2 WT, as shown by biotinylation experiments, suggesting that the Ala270Ser substitution impairs the trafficking and insertion of the transporter to/in the plasma membrane. However, it cannot be excluded that this may be due to different metabolic and efflux activities in cells expressing the two variants of hOCT2.

## 5. Conclusions

In conclusion, the results of this study suggest that the SNP rs316019 of *SLC22A2* may decrease the transporter expression in the plasma membrane and may probably be associated with fewer side effects of the chemotherapeutic treatment with cisplatin. Furthermore, an increased affinity for creatinine of the polymorphic hOCT2 was detected, which may play an important role in the detection of early cisplatin renal injury.

## Figures and Tables

**Table 1:** Summary of apparent affinities (IC_50_ in µM) measured as inhibition of ASP^+^ uptake by hOCT2 WT and hOCT2Ala270Ser using different endogenous and exogenous substances as competitor. The logIC_50_ ± SEM is shown in parenthesis with the degrees of freedom (DF).

Substance	hOCT2 WT IC_50_	hOCT2 Ala270Ser IC_50_
Creatinine	18600 µM (−1.73 ± 0.04; DF = 213)	9800 µM * (−2.01 ± 0.06; DF = 208)
Histamine	4600 µM (−2.33 ± 0.06; DF = 160)	4000 µM (−2.40 ± 0.07; DF = 106)
Serotonin	1900 µM (−2.73 ± 0.15; DF = 46)	1700 µM (−2.78 ± 0.10; DF = 40)
Cimetidine	23 µM (−4.64 ± 0.12; DF = 160)	40 µM (−4.40 ± 0.19; DF = 166)
Cisplatin	16 µM (−4.80 ± 0.10; DF = 34)	9 µM (−5.04 ± 0.11; DF = 40)
Imipramine	2 mM (−2.71 ± 0.06; DF = 238)	1 mM * (−2.89 ± 0.06; DF = 166)
Metformin	868 µM (−3.06 ± 0.05; DF = 178)	848 µM (−3.07 ± 0.05; DF = 178)
Thiamin	87 µM (−4.06 ± 0.05; DF = 160)	106 µM (−3.98 ± 0.06; DF = 172)
Tryptophan	≈ 5400 µM (−2.27 ± 0.28; DF = 40)	≈ 2400 µM (−2.61 ± 0.28; DF = 40)

* indicates a statistically significant difference of the IC_50_ value measured in hOCT2 WT and hOCT2 Ala270Ser cells (extra sum-of-squares F test (GraphPad Prism)).

## Supplementary Materials

The following are available online at https://www.mdpi.com/2218-273X/9/10/578/s1, Figure S1: Representative immunofluorescence analysis of hOCT2 expression, Figure S2: Representative immunofluorescence analysis of negative controls.
