# Supplementary material for: Effects of Single Nucleotide Polymorphism Ala270Ser (rs316019) on the Function and Regulation of hOCT2
Source: Biomolecules. 2019 Oct 7;9(10):578. doi: 10.3390/biom9100578 (PMC6843571; doi:10.3390/biom9100578)
Supplement: Supplementary file 1 [file biomolecules-09-00578-s001.pdf]

Supplementary Materials to

Effects of single nucleotide polymorphism Ala270Ser (rs316019) on the function and regulation of hOCT2

Dominik Frenzel [1], Christina Köppen [2], Oliver Bolle Bauer [2], Uwe Karst [2], Rita Schröter [1], Mladen V Tzvetkov [3] and Giuliano Ciarimboli [1,*]

1 Medizinische Klinik D, Experimentelle Nephrologie, Universitätsklinikum Münster, 48149 Münster, Germany
2 Institut für Anorganische und Analytische Chemie, Westfälische Wilhelms-Universität, 48149 Münster, Germany
3 Institut für Pharmakologie, Abteilung Klinische Pharmakologie, Universitätsmedizin Greifswald, 17487 Greifswald, Germany

* Correspondence: Giuliano Ciarimboli, Universitätsklinikum Münster, Medizinische Klinik D, Experimentelle Nephrologie, Albert-Schweitzer-Campus 1/A14, D-48149 Münster, Germany
gciari@uni-muenster.de; Tel.: +49-251-8356981 Fax: +49-251-8356973

Immunofluorescence analysis of hOCT2 expression in stably transfected HEK cells.

Cells on coverslips were fixed in 4% PFA for 5 min at room temperature. After fixation, nonspecific binding sites were blocked by 1 h incubation at room temperature with 10 % BSA. The cells were then washed with PBS and further incubated overnight at 4°C with hOCT2 antibody [1] diluted 1:20 in 1% BSA. After this, cells were washed three times with PBS, and incubated 45 min at room temperature with the secondary antibody (1:1,000 dilution, Alexa Fluor 488 goat anti-mouse; Invitrogen, Karlsruhe, Germany) together with 4',6-diamidino-2-phenylindol dihydrochloride (DAPI, Thermo Fisher), followed by four more washing steps in PBS. Finally, the cells were covered with Crystal Mount (GeneTex, San Antonio, TX), and evaluated by epifluorescence microscopy (Observer Z1 with apotome; Zeiss, Göttingen, Germany) (Supplementary Material Fig. 1). Negative control slides were included without addition of primary antibody (Supplementary Material Fig. 2).

.

Supplementary material Fig. S1

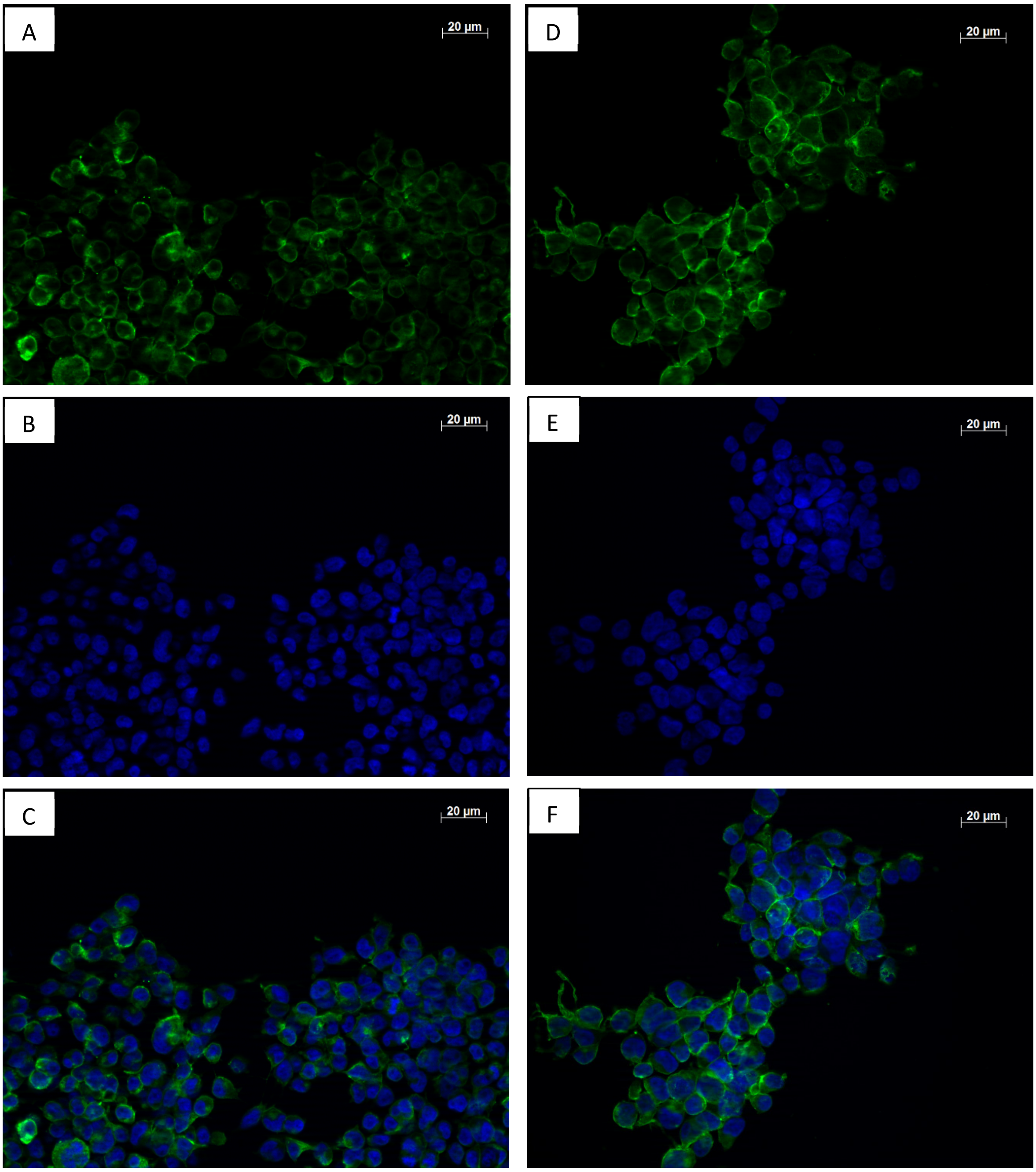

Supplementary Figure S1. Representative immunofluorescence analysis of hOCT2 expression in HEK cells stably transfected with hOCT2 WT (panels A, B, and C) or hOCT2 Ala270Ser (panels D, E, and F). Green is the hOCT2 labelling with a specific antibody against hOCT2 [1]. The cell nuclei are labeled in blue with DAPI. Panels A and D show the fluorescence associated with the hOCT2 antibody, panels B and E the DAPI labelling, and panels C and F the merge picture of hOCT2 and DAPI labelling. As a membrane bound transporter hOCT2 exhibits a typical

predominantly membranous staining on the plasma membrane of HEK cells. The scale bars indicate a length of 20 μm.

Supplementary material Fig. S2

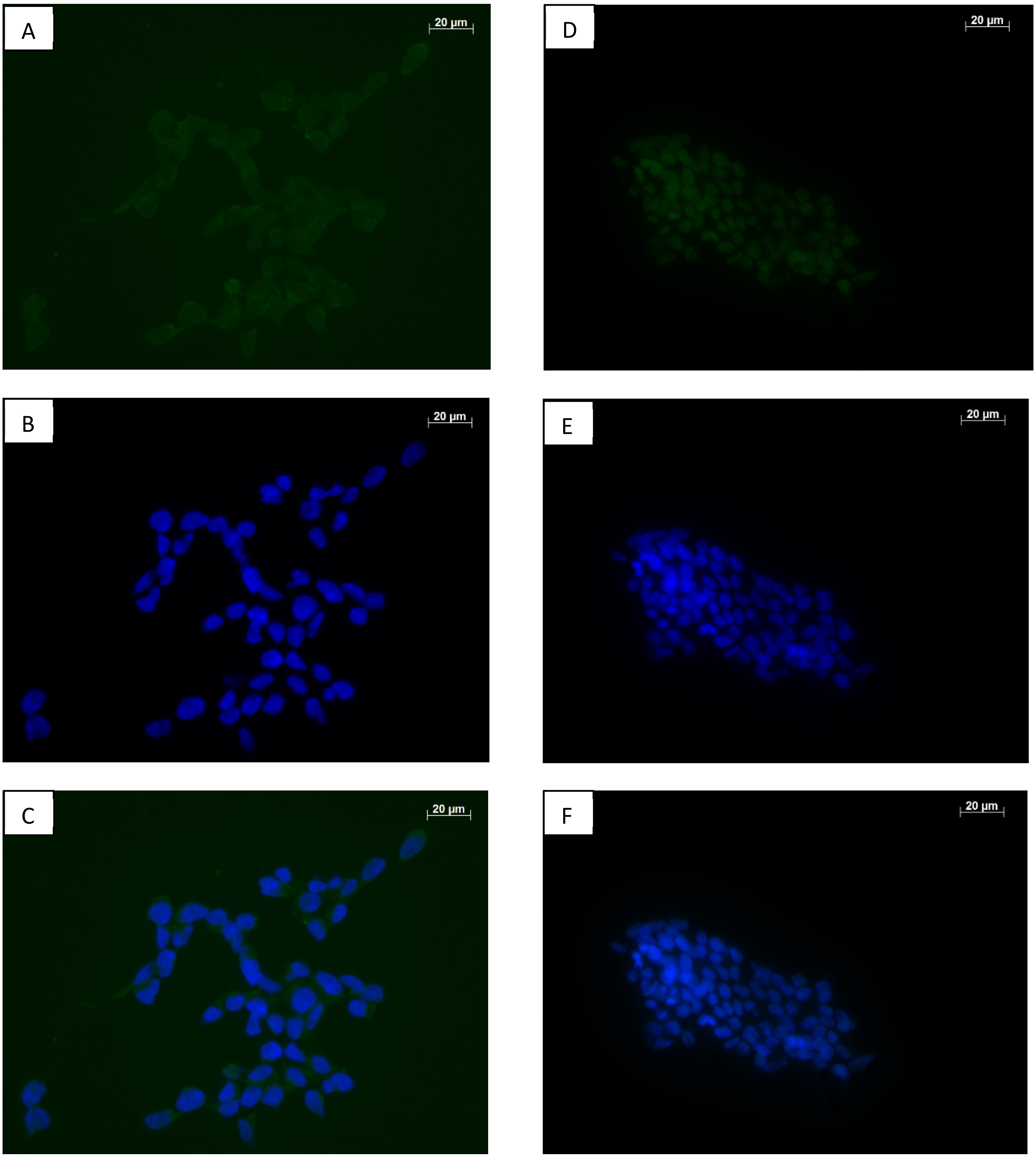

Supplementary Figure S2. Representative immunofluorescence analysis of negative controls for hOCT2 expression in HEK cells stably transfected with hOCT2 WT (panels A, B, and C) or hOCT2 Ala270Ser (panels D, E, and F). Green is the labelling observed using only the secondary antibody for hOCT2 detection. The cell nuclei are labeled in blue with DAPI. Panels A and D show the fluorescence associated with the secondary antibody used for hOCT2

detection, panels B and E the DAPI labelling, and panels C and F the merge picture. The scale bars indicate a length of 20 μm.

Transporter overexpression validated by real-time reverse transcriptase-PCR:

Transfection of the hOCT2-containing constructs in HEK cells resulted in a huge transporter expression as measured in 2 independent clones of hOCT2 WT and 2 independent clones of hOCT2 Ala270Ser, as measured using an real-time PCR assay by Applied Biosystems (for hOCT2 WT: Ct value for hOCT2: 24; Ct value for housekeeping gene (TATA-box binding protein –TBP-): 24; for hOCT2 Ala270Ser: Ct value for hOCT2: 24; Ct value for TBP: 24). The mock transfected cells expressed only minimal amount of hOCT2 (Ct value for hOCT2: 38; Ct value for TBP: 24).

Reference List

1. Biermann, J.; Lang, D.; Gorboulev, V.; Koepsell, H.; Sindic, A.; Schröter, R.; Zvirbliene, A.; Pavenstädt, H.; Schlatter, E.; Ciarimboli, G. Characterization of regulatory mechanisms and states of human organic cation transporter 2. *Am J Physiol Cell Physiol* **2006**, 290, C1521-C1531.
